# Diazepam Photocatalytic Degradation in Laboratory- vs. Pilot-Scale Systems: Differences in Degradation Products and Reaction Kinetics

**DOI:** 10.3390/nano15110827

**Published:** 2025-05-29

**Authors:** Kristina Tolić Čop, Mia Gotovuša, Dragana Mutavdžić Pavlović, Dario Dabić, Ivana Grčić

**Affiliations:** 1 Faculty of Chemical Engineering and Technology, University of Zagreb, Trg Marka Marulića 19, 10000 Zagreb, Croatia; mgotovusa@fkit.unizg.hr; 2 Croatian Meteorological and Hydrological Service, Ravnice 48, 10000 Zagreb, Croatia; ddabic@dhz.hr; 3 Faculty of Geotechnical Engineering, University of Zagreb, Hallerova aleja 7, 42000 Varaždin, Croatia; igrcic@gfv.hr

**Keywords:** diazepam, degradation, photocatalysis, titanium dioxide, hydrogen peroxide, toxicity, pilot-scale process

## Abstract

Industrial growth led to the expansion of existing environmental problems, where different kinds of pollutants can enter the environment by many known routes, particularly through wastewater. Among other contaminants, pharmaceuticals, such as diazepam, once released, pose a significant challenge related to their removal from complex environmental matrices due to their persistence and potential toxicity. For this reason, it is a great challenge to find suitable methods for the treatment of wastewater. The aim of this paper was to investigate the stability of diazepam, subjecting it to various degradation processes (hydrolysis and photolysis), focusing on photocatalysis, an advanced oxidation process commonly used for the purification of industrial wastewater. The photocatalytic system consisted of UV-A and simulated solar irradiation with titanium dioxide (TiO_2_) immobilized on a glass mesh as a photocatalyst, with an additional reaction performed in the presence of an oxidizing agent, i.e., hydrogen peroxide, to improve diazepam removal from water matrices. The kinetic rate of diazepam degradation was monitored with a high-performance liquid chromatograph coupled with a photodiode array detector (HPLC-PDA). The target compound was characterized as a hydrolytically and photolytically stable compound with t_1/2_ = 25 h. The presence of an immobilized TiO_2_ catalyst contributed significantly to the degradation of diazepam under the influence of UV-A and simulated solar radiation, with t_1/2_ in the range of 1.61–2.56 h. Five degradation products of diazepam were identified at the laboratory scale by MS analysis (*m*/*z* = 267, *m*/*z* = 273, *m*/*z* = 301, *m*/*z* = 271, and *m*/*z* = 303), while the toxicity assessment revealed that diazepam exhibited developmental toxicity and a low bioaccumulation factor. The pilot-scale process resulted in significant improvements in diazepam degradation with the fastest degradation kinetics (0.6888 h^−1^). These results obtained at the pilot scale highlight the potential for industrial-scale implementation, offering a promising and innovative solution for pharmaceutical removal from wastewater.

## 1. Introduction

This paper is mainly concerned with the active pharmaceutical compound diazepam, a member of the group known as benzodiazepines, which are potential new harmful substances. These psychotropic drugs can contribute to muscle relaxation and relief of anxiety, insomnia, panic attacks, phobic disorders, seizures, and alcohol withdrawal symptoms by altering the action of the neurotransmitter gamma-aminobutyric acid (GABA) [1,2]. The importance of studying these drugs, especially diazepam, when released into the environment, lies in their increasing use over the years. Due to the increasing use of diazepam and other drugs over the years, the importance of studying their fate and behavior in the environment is highlighted, as they are present in various water matrices around the world. In general, benzodiazepines are one of the most commonly prescribed drugs in the world, with diazepam, commonly marketed under the trade name Valium, being widely used from its approval in 1963 to the present day, with more than ten million prescriptions per year [2,3,4].

Accordingly, it is not surprising that diazepam enters the environment through various pathways (the excretion of the active ingredient and metabolites, industrial and medical waste, and wastewater) [5,6]. Past research has shown that both direct and indirect photolysis represent a significant removal process of diazepam and its 14 identified photoproducts [7] in the photic zone of the aqueous environment. However, diazepam, like other psychotropic drugs, tends to be detected in the tissues of aquatic organisms, so its presence in surface waters is very likely. In fact, diazepam has been detected at concentrations ranging from 1.9 to 1180 ng/L in various water matrices, such as sewage treatment plant influent and effluent, sludge, rivers, surface water, and even drinking water [5,8,9], urging the necessary improvements in water treatment techniques. Diazepam is metabolized in the human body to temazepam, nordiazepam, and oxazepam, whose persistence in surface waters has also been confirmed worldwide [8,10].

The modernization of technology and science with a low level of regulation has resulted in the release of many agents with pseudo-persistent properties into the environment, which poses a direct threat to our limited drinking water supplies [9,11,12,13,14,15]. Aware of the problem of water scarcity, scientists nowadays pay much attention to the development of appropriate methods for the treatment of wastewater, which is the main source of pharmaceuticals and other small organic pollutant molecules in various environments [16,17,18]. Since conventional water treatment methods are ineffective in removing pharmaceuticals, the development of advanced oxidation processes has shown great promise for water purification by generating non-selective, highly oxidative species, i.e., hydroxyl radicals. The use of a photoactive semiconductor, usually TiO_2_, a chemical, thermally stable, and environmentally safe material, and a light source—a process known as heterogeneous photocatalysis—has been the focus of pharmaceutical degradation for many years [19,20,21,22,23,24]. In general, during photocatalysis, the surface of a semiconductor material is activated by absorbing photon energy equal to or greater than the gap energy, resulting in the excitation of electrons and the formation of electron–hole pairs, which undergo photoreduction and photo-oxidation reactions with electron-acceptor and electron-donor molecules, creating reactive oxygen species (primarily ·OH radicals) that non-selectively oxidize and degrade different organic pollutants [25,26,27]. Despite the good characteristics of TiO_2_, which is why it is most commonly used in AOPs, the biggest drawback is the energy contribution due to the UV radiation limit and the possibility of recombination [28,29]. In addition, a hydrogen peroxide combined TiO_2_ system can improve the limited degradation efficiency due to the promoted formation of hydroxyl radicals [30].

In this research, diazepam was subjected to photolytic and photocatalytic degradation to reveal the mechanism of its removal from water, since biodegradation and mineralization were slow, as previously published, [7]. In the reaction system, titanium dioxide was used as a catalyst layer immobilized on prepared glass mesh substrates instead of the commonly tested suspension form to simplify performance and reduce the cost [31,32,33]. Although most degradation studies are carried out at a laboratory scale, there is growing interest in the application of photocatalysis at a larger scale, specifically to investigate the efficiency, reactor design, and viability, ultimately leading to its application in industry [34]. Therefore, in this paper, in addition to photocatalysis carried out at a laboratory scale under UV-A light, a further degradation experiment was also carried out, but at a pilot scale using an artificial source of solar radiation.

It was taken into account that, by reducing the concentration of the target compound through the degradation process, more polar, toxic, and, therefore, more environmentally harmful substances may be produced. Since the pharmaceuticals act in certain biological systems, the ecotoxicological effects on the biota and the uncontrolled environment should also be investigated. To complete our knowledge on the effects of pharmaceuticals on the environment, different organisms are used to assess the acute and chronic toxicity of pharmaceuticals. Diazepam showed adverse effects on *Daphna magna*, zebrafish *Danio rerio embryos*, mussel *Elliptio complanate*, etc. [10,13].

Therefore, samples were collected during diazepam degradation and monitored by HPLC-PDA and HPLC-MS to identify the degradation products and, finally, estimate the toxicity using *Vibrio fischeri* and T.E.S.T. software version 4.2.1. based on QSAR methods.

## 2. Materials and Methods

### 2.1. Materials and Reagents

Diazepam (DIA) with a purity of > 99.9% was obtained from JGL, d.d. (Rijeka, Croatia). This pharmaceutical with the chemical name 7-chloro-1,3-dihydro-1-methyl-5-phenyl-2H-1,4-benzodiazepin-2-one and a molecular weight of 284.75 g/mol is a poorly water-soluble compound with log *K*_ow_ and p*K*_a_ constants of 2.8 and 3.4. The standard solution of DIA was prepared by weighing an exact amount of the standard, and then dissolving it in 1% methanol (HPLC, J. T. Baker, Deventer, The Netherlands) and Milli-Q water to obtain final concentration of 10 mg/L.

Photocatalytic experiments were performed with a TiO_2_ suspension (Aeroxide^®^ P25, Evonik, Essen, Germany) immobilized on a glass mesh adjusted to the size of the reactor. The sol–gel method was applied according to the procedure described in previous publications and the material was characterized by SEM analysis, Raman spectroscopy, and energy-dispersion spectroscopy (EDX) [35,36,37,38,39]. The mass of the TiO_2_ layer produced was 0.2016 ± 0.055 g. The mass of the dry glass mesh was checked regularly to ensure that the same amount of photocatalyst was present in each degradation process.

Other chemicals used in the experimental part of this work were acetonitrile (HPLC grade, J. T. Baker, The Netherlands), formic acid (p.a., T. T. T. d.o.o., Sveta Nedjelja, Croatia), and hydrogen peroxide (30%, Gram-mol d.o.o., Zagreb, Croatia).

### 2.2. Degradation Experiments

All laboratory-scale degradation experiments were carried out in an open rectangular batch reactor made of Plexiglas with the dimensions 17.5 × 4.6 cm and a working volume of 100 cm^3^. The continuous flow rate of 30 mL/min was achieved with a peristaltic pump. The source of simulated solar radiation was a Philips 8 W UV-A tube lamp with E_max_ 365 nm, which was positioned 7 cm above the reactor. At this position, the UV-A intensity was determined using a radiometer, with an average value of 0.75 mV/cm^2^. In order to illuminate the reactor chamber uniformly, the lamp was covered with aluminum foil during all experiments, which served as a reflective surface.

All experiments were carried out at room temperature. At specific time intervals, 500 μL were sampled and analyzed by HPLC-PDA. The degradation experiments included the following: photolysis of the DIA solution (UV lamp only), photocatalysis (TiO_2_ glass mesh placed in the reactor + UV lamp), and photocatalysis experiments with the addition of H_2_O_2_. In addition, diazepam photocatalysis was carried out in wastewater instead of Milli-Q water to determine the influence of the water matrix. The wastewater sample was collected in October 2021 in Italy at the influent of a wastewater treatment plant as a mixture of municipal and hospital wastewater (pH 6.88, conductivity 868 μS/cm, COD 182 mg/L O_2_, BOD 90 mg/L O_2_, DOC 18.5 mg/L, TSS 82 mg/L, N_tot_ 31 mg/L, and P_tot_ 6 mg/L). Before use, this sample was sterilized and filtered to avoid microbiological effects. Preliminary experiments were also performed without UV lamp for each degradation experiment to exclude any change in DIA concentration due to adsorption on a TiO_2_ support. The hydrolysis experiments were carried out according to the OECD method [40].

A flat-plate cascade reactor (FPCR) was used to study the photocatalytic degradation of targeted pollutants at a pilot scale, as it represents a realistic outdoor system [41].

The modular FPCR reactor consisted of three cascades (250 × 500 mm each) made of resistant PMMA, a collecting tank, a Masterflex peristaltic pump, and a panel with full-spectra lamps. The distance between the cascade surface and the center of the lamps were 7 cm at the inlet and 2 cm at the outlet position to allow for the cascade length and the effective lamp length. The water was continuously recirculated from the collecting tank through the cascades with the flow rate set to *Q*_1_ = 13.0 cm^3^ s^−1^. The total volume in the recirculation was 2.0 L in each case, respectively. The depth of the water layer (h) was 0.6 ± 0.1 cm, respectively. Full spectra lamps were used: JBL SUN GLO (various COLOR, TROPIC, and NATUR models) (T5 types, length 145 cm, and rated power 80 W). The UVB and UVA intensities at the lamps wall (*I*_w_) and the distance from the photocatalyst surface (*I*_0_) were determined radiometrically using the UVX radiometer, which was equipped with the corresponding UV-B sensor UVX-31 (medium range 280−340 nm) and the long-wave UV-A sensor UVX-36 sensor (range 335–385 nm) with a sensor accuracy of ±5% (UVP Products, Cambridge, UK). Details of the arrangement of the lamps on the plate and the respective intensities measured at the wall lamps and the photocatalyst surface can be found in a previous publication [41].

### 2.3. Analytical Procedure

Analysis of standards and residues was monitored using the HPLC-PDA instrument (Waters 2795 Alliance HPLC System with 2996 PDA detector, Milford, MA, USA), while chromatographic separation was performed using a 150 × 4.6 mm Kinetex C18 column (Santa Clara, CA, USA) with a particle size of 5 μm. The mobile phase consisted of 55% of 0.1% formic acid in Milli-Q water as eluent A and 45% of 0.1% formic acid in acetonitrile as eluent B in isocratic elution mode for 20 min. The flow rate was 0.5 mL/min with injection volume of 20 μL. Diazepam was detected in the chromatograms obtained based on its retention time of 10.04 min with an absorption maximum at a wavelength of 231 nm. The degradation products were confirmed by HPLC-MS (Agilent 1200 in conjunction with triple quadrupole Agilent 6410, Santa Clara, CA, USA) under the identical chromatographic conditions described above.

### 2.4. Acute Toxicity Assessment

Bioluminescent bacteria *Vibrio fischeri* was used to test the acute toxicity of a standard solution of diazepam (10 mg/L). Experiments were performed by measuring the inhibition of luminescence before and after 30 min of contact between serially diluted DIA samples with 2% NaCl and a bacterial suspension on a luminometer (LUMIStox 300 Hach Lange, Düsseldorf, Germany) at 15 °C. A detailed procedure is described in the previously published paper [42].

## 3. Results and Discussion

By conducting the experiment in accordance with the OECD guideline [40], the possibility of diazepam hydrolysis was first investigated. This guideline describes a laboratory test method to evaluating the abiotic hydrolytic conversion of chemicals in aqueous systems at the pH values commonly found in the environment (pH 4–9). Experiments are performed to determine the hydrolytic stability of the test substance as a function of pH. In similar studies, the possible degradation pathways of diazepam in alkaline medium were determined [43]. The results of these analyses showed the stability of diazepam when exposed to the aquatic environment with hydrolysis < 10% (Figure S1). This means that a decrease in the concentration of this pharmaceutical can be explained exclusively by the influence of other factors, such as the UV-light used.

Previous studies on the kinetics of diazepam degradation in the presence of various external conditions (e.g., active sludge) have shown that the reactions follow the first-order reaction model [44]. The same observation can be made in this case, where the first-order reaction model is described by the following equation [45]:

(1) $$\ln\left(\frac{c_{0}}{c}\right)=-k\cdot t$$

where *c*_0_ is the initial concentration of DIA and *c* is the concentration DIA at the time of sampling. The first-order rate constant, *k*, obtained from the slope of ln(*c*_0_/*c*) versus *t* is used to calculate the half-life of DIA according to the following equation:

(2) $$t_{1/2}=\frac{\ln2}{k}$$

The values of the reaction order constants, half-life, and correlation factor obtained from each experiment are listed in Table 1.

The basic kinetic equation presented was used to compare the degradation kinetics in different applied processes. More complex kinetics are presented in one of the following subsections.

In all degradation experiments, the pH of the solution DIA was not adjusted, but only controlled before and after degradation. The initial solutions had pH values in the range of 4.8–5.2, while the final pH values depended on the degradation efficiency and were in the range of 3.1–3.8. In the preliminary experiments, where no degradation was observed, the pH of the DIA solution was close to the initial value.

### 3.1. Photolysis of DIA

**Figure 1:**
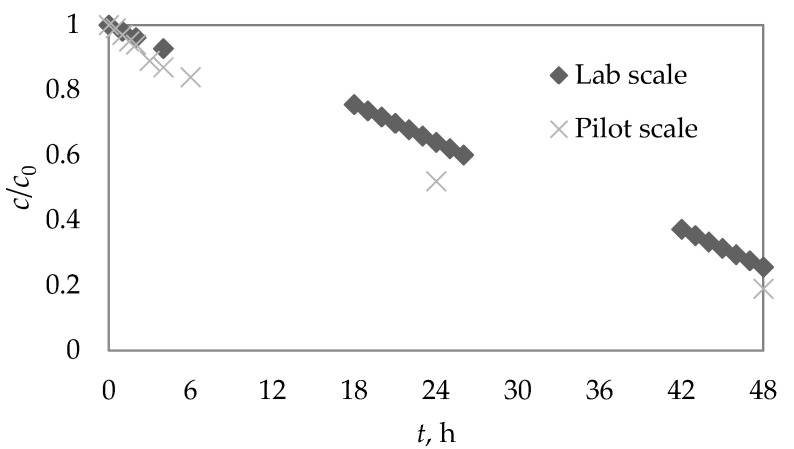
Photolysis of DIA following the first-order reaction model.

The DIA standard solution in ultrapure water was irradiated with UV-A and artificial solar irradiation for 48 h (Figure 1).

During the irradiation period, 75% of the DIA was degraded with a half-life of 25.1 h. Compared to previous studies [7], it can be noted that DIA in water has a half-life of about 100 h, which can be attributed to the effect of the water matrix whose substances can delay photodegradation [46,47,48]. On the other hand, indirect photolysis by the addition of humic acids in the model solution decreased the half-life to 28 ± 12.2 h, indicating a significant effect of dissolved organics on the removal of this pharmaceutical. The absorption spectra of the DIA solution showed a maximum at 231 nm, which is not close to the dominant region of UV-A radiation at 365 nm. Due to the insufficient energy of the irradiation source used, it is logical that the degradation of the organic compound DIA was slow. The UV-B irradiation by full spectra lamps provided additional energy but the resulting photolysis was also slow.

### 3.2. Photocatalytic Degradation of DIA at Laboratory Scale

In the preliminary experiments without UV irradiation, no significant decrease in DIA concentration was observed due to the adsorption on TiO_2_, so that a sorption/desorption equilibrium was established for 30 min prior to illumination. After TiO_2_ activation under light, the DIA concentration was reduced by 89.3% within 8 h, leading to an increase in the reaction rate constant and a significant decrease in the corresponding half-life. Similar results were obtained in other studies [49], according to which the impregnation of commercial TiO_2_ on borosilicate glass beads led to an 88% degradation of DIA, which is higher than the amount observed only in photolysis performed under the same conditions. Comparing the *k* values of direct photolysis and photocatalysis for the DIA degradation (0.0276 h^−1^ and 0.2691 h^−1^, respectively), it can be concluded that the introduction of the catalyst in the degradation process plays an important role in the removal of DIA from water. A similar trend between the photolytic and photocatalytic experiments was previously observed for ibuprofen [50]. The influence of the water matrix on the photocatalytic removal of DIA was also determined by performing the reaction in the wastewater sample. As can be seen (Table 1), the DIA removal was slowly retarded from 0.2691 to 0.2037 h^−1^, indicating the broad applicability of the tested photocatalytic system, despite a more complex matrix (metals, various inorganic salts, organic compounds including other emerging impurities, etc.). The reduction in the kinetic rate of DIA removal in wastewater can also be explained by the increase in the pH of the solution compared to the reaction carried out in Milli-Q water, where the pH was slightly acidic [51].

### 3.3. Effect of Addition of H_2_O_2_

**Figure 2:**
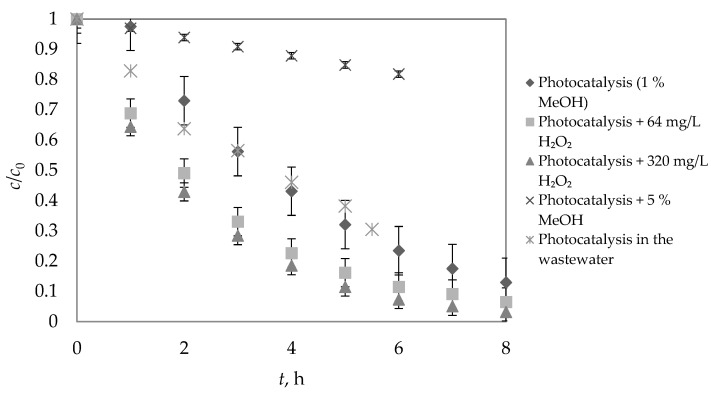
Time profile of DIA degradation in laboratory scale reactor (error bars refer to relative standard deviation, *n* = 2).

The next series of experiments involved the UV/TiO_2_ degradation of DIA with the addition of an electron acceptor to the reaction solution at the beginning of photocatalysis to achieve concentrations of 64 and 320 mg/L. H_2_O_2_ as a strong oxidizing agent in the presence of UV light can accelerate the formation of additional hydroxyl radicals and promote the catalytic process of the organic compound [52]. Both effects, positive or negative, are known for the addition of H_2_O_2_ to the reaction solution; therefore, DIA photocatalysis was performed at two concentration levels. The results showed (Figure 2) that a lower H_2_O_2_ concentration led to a lower amount of hydroxyl radicals formed in the system. A higher H_2_O_2_ concentration led to an increase in the constant oxidation rate with no inhibitory effect.

In general, the presence of H_2_O_2_ contributes to the degradation rate of DIA compared to the photocatalytic treatment without a peroxide addition, achieving 93.5 and 96.8% of degradation (Table 1).

The increased photocatalytic rate of DIA removal due to the synergistic effect of the semiconductor material and the oxidizing agent is explained by the promotion of ·OH radicals presented by few reactions; peroxide undergoes direct photolysis when the UV light source is applied (Equation (4)) and can also act as an electron scavenger and prevent recombination in TiO_2_ (Equation (3)) [28,53]:


H_2_O_2_ + e^−^(cb) → ·OH + OH^−^ (3)

(4) $$H_{2}O_{2}\overset{hv}{\to }2\cdot OH$$

When combined with a semiconductor photocatalyst, the decomposition of H_2_O_2_ is more efficient, as reported by Shiraishi et al. and Rosa et al. [54,55].

### 3.4. Effect of Methanol on Diazepam Photocatalysis

The mechanism of photocatalytic degradation of DIA was determined by studying the same oxidation process, only with a different percentage (1% and 5%) of methanol added during the preparation of the standard solutions.

To explain the results shown in Figure 2, the presence of alcohol in the system can be associated with the scavenging of hydroxyl radicals. The higher proportion of MeOH in the solution led to a delay in the degradation of DIA. Since MeOH was found to scavenge OH radicals and holes, the photocatalytic mechanism of DIA degradation plays an important role for these two mentioned reactive species. They are formed by the excitation of TiO_2_ and the transfer of electrons from the valence band to the conductive band, whereby the cavities on the hydrated and hydroxylated surface of the titanium dioxide retain the hydroxyl radicals bound to it [56,57]. Moreover, in addition to the sparing effect of hydroxyl radicals, MeOH can also oxidize and form hydroxymethyl radicals in the TiO_2_/*hν* system, and furthermore, the reduced DIA removal can also be explained by the ability of MeOH to adsorb on semiconductor material, which acts as a hole scavenger [58,59].

### 3.5. Identification of Photolytic and Photocatalytic Degradation Products of Diazepam

**Figure 3:**
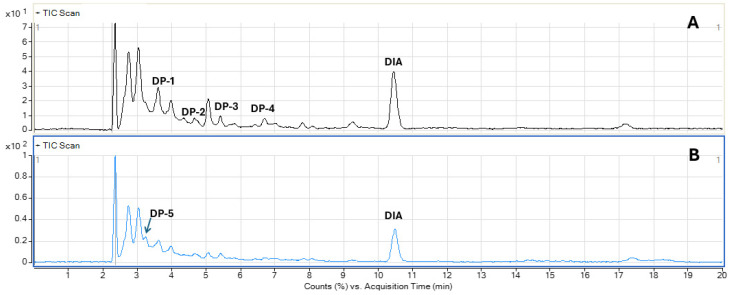
Identification of DPs during photocatalysis without (**A**) and with addition of hydrogen peroxide (**B**).

To observe the time profiles of DIA degradation products by HPLC-PDA, DIA was photodegraded in a solution of 10 mg/L by the photolytic and photocatalytic process. A comparison of the chromatograms observed during the photocatalytic experiments with the chromatogram of the diazepam before degradation shows the formation of five degradation products with *m*/*z* 273 (DP-1), 303 (DP-2), 301 (DP-3), 271 (DP-4), and 267 (DP-5) (Figure 3). The lower retention times of all five degradation products compared to diazepam indicate their higher polarity. The degradation of DIA was confirmed and the preliminary structures of the DPs were proposed by HPLC-MS. The mass spectra used to identify each of the degradation products are listed in the Supplementary Materials (Figures S1–S5).

**Scheme 1:**
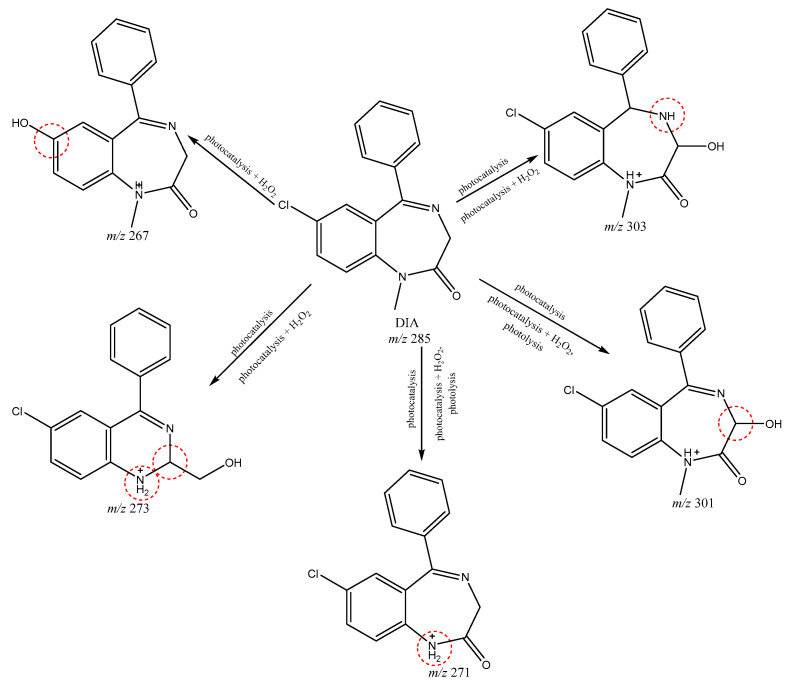
Proposed degradation pathway of the diazepam.

Photocatalysis gave the same four degradation products (with *m*/*z* 273, 301, 271, and 303) when peroxide was not used (Scheme 1). When peroxide was added to the reaction system, the fifth degradation product appeared, DP-5 with *m*/*z* = 267. Finally, only two degradation products were observed in the photolytic experiments (DP-3 and DP-4).

Considering the activity of the hydroxyl radicals involved in the reaction mechanism, five main degradation products were formed by oxidation reactions via OH-addition, H-abstraction, and electron transfer [60]. DP-4 and DP-3 as a result of the *N*-demethylation and C-hydroxylation of a heterocyclic ring of the analyte correspond to the two diazepam metabolites nordiazepam and temazepam, respectively. DP-2 with *m*/*z* 303 are also the result of the reactions of demethylation and the hydroxylation reaction of the parent compound, which is also known as another DIA metabolite—oxazepam. The similarity of DIA degradation by AOPs and the DIA metabolic pathway in humans was also confirmed by a study published in 2017 [61]. Based on the spectra obtained, DP-1 with *m*/*z* 273 is described as (6-chloro-4-phenyl-1,2-dihydroquinazolin-2-yl)methanol—a degradation product, which has already been published in the literature [7,62]. Photocatalysis with the addition of peroxide led to the formation of DP-5 with *m*/*z* 267with a possible mechanism of the dechlorination and hydroxylation of DIA, resulting in a hydroxylated derivative.

**Figure 4:**
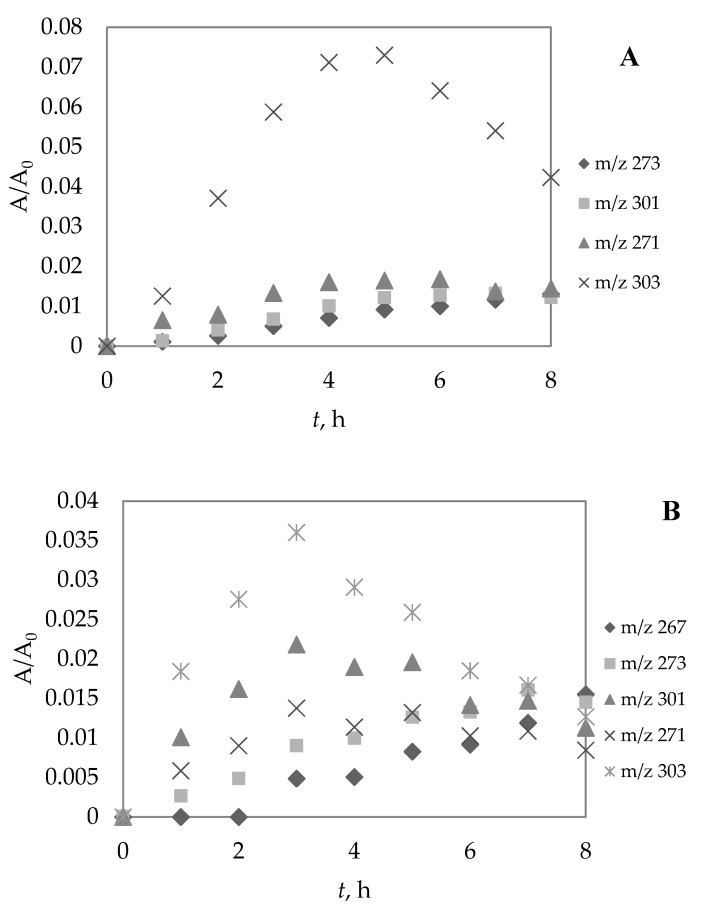
Degradation profiles of formation of five degradation products during photocatalysis (**A**), and photocatalysis with H_2_O_2_ addition at a concentration of 64 mg/L (**B**).

In the absence of suitable standards, the DP profiles were plotted using the normalized peak area (A/A_0_; A_0_—initial concentration of DIA) for each degradation product at a particular time point during a given photodegradation process (Figure 4).

The degradation products formed by photocatalysis reached their maximum concentration after 4 h, and none of them was completely degraded within the process time of 8 h. During the irradiation of the reaction system to which the oxidizing agent was added, most DPs showed the highest intensity after 3 h of photocatalysis, while the concentration of the newly formed DP with *m*/*z* 267 increased steadily during the process time.

### 3.6. Photocatalytic Degradation of DIA at Pilot Scale

**Figure 5:**
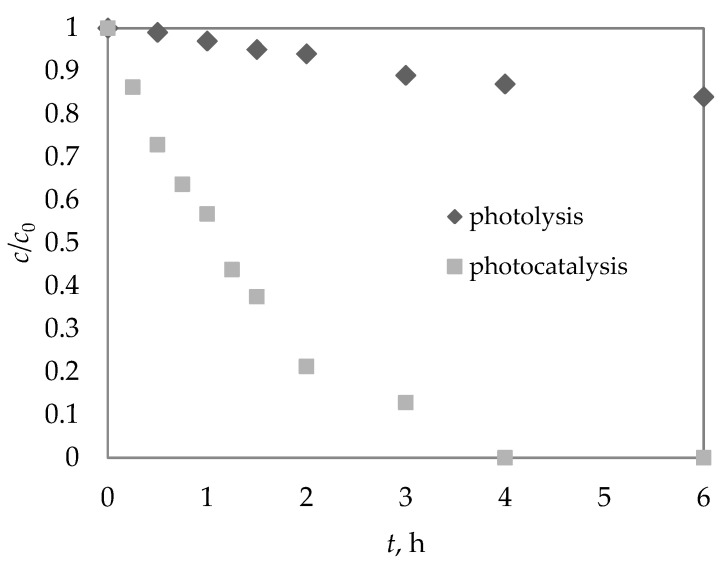
DIA degradation kinetics at pilot scale.

The degradation kinetics of DIA during photocatalysis in FPCR is shown in Figure 5. The results suggested the importance of applied irradiation. As given in Table 1, the observed first-order reaction rate constants in the pilot-scale FPCR was 0.6888 h^−1^, which is almost three times higher than the observed constant at the laboratory scale.

The basic kinetic equations were modified to give more intrinsic parameters related to the photocatalytic degradation of DIA over the irradiated TiO_2_ film. Incident photon flux was introduced to the basic kinetic equation:

(5) $$r_{\mathrm{DIA}}=\frac{dC_{\mathrm{DIA}}}{dt}=-k_{i}{\left({\left(\mu I_{0}\left(T,H\right)\right)}_{\mathrm{UVB}}+{\left(\mu I_{0}\left(T,H\right)\right)}_{\mathrm{UVA}}\right)}^{m}{\left(C_{\mathrm{DIA}}\right)}^{n}$$

The *I*_0_(T,H) (W m^−2^) stands for the incident photon flux at the film surface along the cascade width and length, *μ* (m^−1^) is the absorption coefficient averaged over the spectrum of incident irradiation (in the UVB and UVA region), and m is the order of the reaction with respect to irradiation absorption. Note that *k*_i_ stands for the intrinsic degradation rate constant of DIA. The full modeling approach was given in a previous publication [41].

The modified kinetic model was applied to both the laboratory- and pilot-scale experiments and the results are given in Table 2.

A similar intrinsic reaction rate constant was determined for both reactors, namely, 3.65 × 10^−5^ and 3.61 × 10^−5^ h^−1^ W^−0.5^ m^1.5^, respectively, which confirms the major influence of irradiation on DIA degradation kinetics.

**Figure 6:**
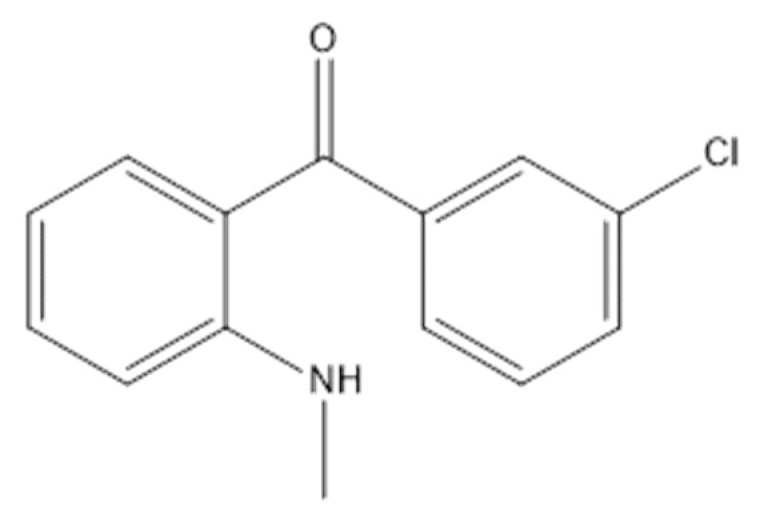
Tentative structure of degradation product identified at pilot scale.

Fewer degradation products were observed during pilot-scale photocatalysis. Three degradation products were identified: the previously presented products with *m*/*z* 271 and 301 and a new degradation product with *m*/*z* 246. The latter was also presented in the literature as a DIA degradation product [61] and identified as 9,5-chloro-2-(methylamino) benzophenone (Figure 6). The observed degradation products primarily correspond to the hydroxylation of DIA after the attack of the ·OH radical, but further oxidation with the opening of the diazepine ring was also observed.

### 3.7. Reusability of the Photocatalyst

**Figure 7:**
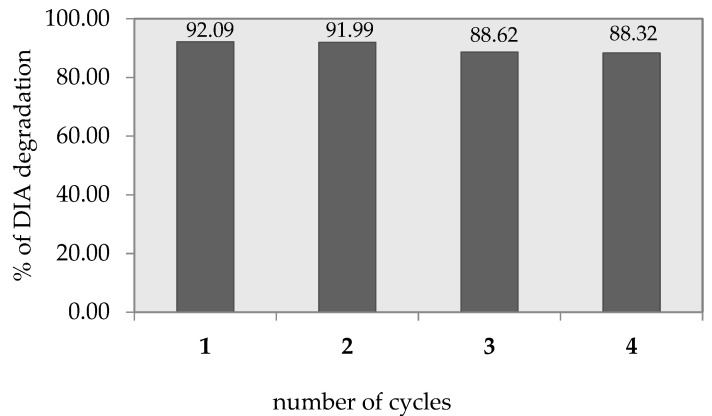
Degradation efficiency after four cycles of DIA photocatalysis.

To evaluate the reusability of the immobilized TiO_2_ photocatalyst, four consecutive cycles of DIA photocatalysis were performed under the same experimental conditions (10 mg/L and 8 h of irradiation). After each cycle, the catalyst was washed with water, dried, and reintroduced into a freshly prepared DIA standard solution. As shown in Figure 7, the photocatalytic efficiency slowly decreased with each cycle. After four consecutive cycles of photocatalysis, the overall efficiency decreased by about 3.7%, indicating the good stability of the immobilized material.

### 3.8. Toxicity Assessment

The bioluminiscence method for determining the toxicity of DIA is based on the use of the bacteria *Vibrio fischeri*. The growth of these bacteria is inhibited when they are in an environment containing organic pollutants. In other words, it is possible to determine the acute ecotoxicity of a particular pharmaceutical using this method by reducing the natural luminescence of the bacteria mentioned. The method used in this paper to determine toxicity was the German standard method DIN 38 412-L34, which takes 30 min [63].

**Figure 8:**
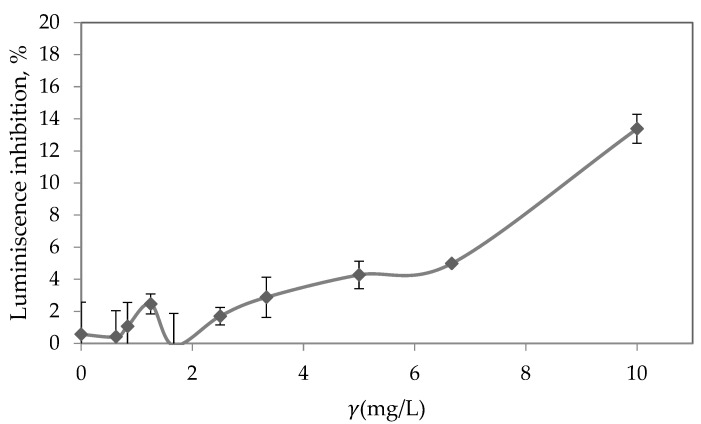
Dependence of luminescence inhibition on the concentration of DIA (error bars refer to standard deviation, *n* = 2).

The experimental results obtained to evaluate the toxicity of DIA show that up to 13.4% were inhibited over a period of 30 min. From this, it can be concluded that the required DIA concentrations would have to be higher than 10 mg/L to inhibit 20% of the *Vibrio fischeri* bacterial culture (Figure 8).

Another way to determine the toxicity of DIA is to use the Toxicity Estimation Software Tool (T.E.S.T.) version 4.2.1. in combination with the Consensus method, which was used to evaluate the bioaccumulation factor and developmental toxicity of DIA (Table 3).

The bioaccumulation factor is the ratio between the concentration of a given chemical in the fish as a result of absorption by the surfaces of the respiratory system and the concentration of the chemical in the water in a steady state. The developmental toxicity parameter, on the other hand, indicates whether or not a particular chemical causes developmental toxic effects in humans or animals. The software results predicted that DIA should have a low bioaccumulation factor (less than 30; 26.64), which also indicates developmental toxicity (+(0.83)) [64]. According to the nearest neighbor method, all DPs are estimated to have a positive developmental toxicity potentially higher than that of the parent compound, which is the most common problem when byproducts may have greater toxicity. The original compound, diazepam, also has a negative mutagenicity −(0.00) according to the Consensus method. Besides this degradation product, *m*/*z* = 273 is the only compound obtained that has a known value for the bioaccumulation factor value, 87.84 by the nearest neighbor method. Compared to DIA, this is a much higher value that falls in the range of intermediate values (between 30 and 100) [65]. Toxicity results are also described by interpreting the results estimated for species commonly used for environmental and human risk assessment. These predictions indicate that DIA and DP pose a similar acute toxicity risk to freshwater invertebrates (with the exception of *m*/*z* 267 with a lower value for *Daphnia magna* but a higher risk to mammals). The results obtained indicate that further experimental validation of the toxicity results is required.

### 3.9. Comparison of Diazepam Removal with Other Studies

Table 4 contains a comparison of different degradation methods applied for the removal of DIA, a non-biodegradable compound and the target analyte of this study. To the best of the author’s knowledge, there are few published methods that specifically address the removal of DIA, particularly those that compare the laboratory- and pilot-scale performance. As shown, DIA is very photolytically stable and has long degradation half-lives. This emphasizes the need for the further investigation of alternative degradation methods, such as advanced oxidation processes and membrane technologies. These techniques have shown a high removal efficiency for DIA compared to the original drug concentration. In particular, the photocatalysis method investigated in this paper yielded promising results and achieved improved degradation at a pilot scale. These results suggest the possibility of scaling up to industrial applications using environmentally friendly and cost-effective materials such as immobilized TiO_2_.

## 4. Conclusions

In this paper, a series of experiments were performed to investigate the possibility of photocatalysis using immobilized titanium dioxide to degrade the hydrolytic and photolytic stable molecule diazepam. In particular, photocatalysis experiments were carried out in ultrapure water, and then with the addition of hydrogen peroxide at a laboratory scale and compared with pure photocatalysis at a pilot scale. From the results obtained, it can be concluded that the kinetics of diazepam removal follows a first order with *R*^2^ greater than 0.9570 for all degradation methods. While photolysis removed diazepam from the water only to a small extent, the addition of TiO_2_ in the reaction system accelerated the degradation from 74% in 48 h to 88% in 8 h of illumination. The addition of oxidizing agents to the solution promoted DIA degradation, with the fastest degradation occurring with a higher concentration of H_2_O_2_ (*t*_1/2_ 1.62 h) The addition of alcohol drastically decreased the rate constant of photocatalysis from 0.2691 to 0.0334 h^−1^, demonstrating the important role of hydroxyl radicals in DIA degradation, which attacked the DIA heterocyclic ring of DIA and formed a total of five degradation products through, mainly, the reaction of demethylation and hydroxylation. The immobilized photocatalyst showed a good reusability of up to four cycles, indicating a cost-effective and environmentally friendly material for further scale conversion. Diazepam inhibited 13.4% of the bacterial culture of *Vibrio fischeri*. The toxicity of the degradation products was compared with that of the parent molecule based on characteristics estimated using with the T.E.S.T. software. All tested molecules showed a positive developmental toxicity, even higher than that of diazepam, while the product with *m/z* 273 potentially has a high tendency to bioaccumulate. Finally, photocatalysis has been shown to be a good way to reduce the concentration of potentially toxic organic compounds such as pharmaceuticals. Due to its lower energy consumption compared to UV-C light, it has the potential to be used on a larger scale. In addition, it can be used as a natural part of the solar radiation that reaches the earth’s surface. Pilot-scale experiments showed an improved efficiency of DIA degradation even without additional oxidizing agents (with a half-life of 1 h). The utilization of solar radiation leads to faster reaction kinetics and less harmful degradation products, confirming its applicability in the future with lower costs and improved TiO_2_-based materials.

## Figures and Tables

**Table 1:** Comparison of the results of kinetic analysis of all observed experimental systems (values in brackets refer to relative standard deviation (*n* = 2)).

	*t*_1/2_, h	*k,* h^−1^	*R* ^2^
Photolysis	25.11	0.0276 (±0.0026)	0.9570
Photocatalysis	2.58	0.2691 (±0.12)	0.9884
Photocatalysis + 64 ppm H_2_O_2_	2.02	0.3433 (±0.047)	0.9969
Photocatalysis + 320 ppm H_2_O_2_	1.61	0.4298 (±0.030)	0.9996
Photocatalysis in wastewater	3.40	0.2037	0.9650
Photocatalysis + 5% MeOH	20.75	0.0334 (±0.011)	0.9992
Pilot-scale photocatalysis	1.01	0.6888	0.9908

**Table 2:** Results of kinetic model using Equation (5).

	Laboratory Scale Photocatalysis	Pilot-Scale Photocatalysis in FPCR
μ_UVB_, m^−1^	2.0 × 10 [36,41]	
μ_UVA_, m^−1^	7.3 × 10^6^	
*I*_0, UVA_, Wm^−2^	7.5	15.4 *
*I*_0, UVB_, Wm^−2^	0	3.6 *
*k*_i_, h^−1^ W^−0.5^ m^1.5^	3.65 × 10^−5^	3.61 × 10^−5^

* Average values; *I*_0_ values along cascade length and width were used for calculations.

**Table 3:** Toxicity prediction of DIA and DPs formed during photocatalysis.

Analyte	Developmental Toxicity	BAF Log 10	Mutagenicity	Daphnia Magna LC50 (48 h), mol/L	Oral Rat LC50 (48 h) mol/kg	Fathead Minnow LC50 (96 h) mol/L	Method
DIA	+(0.83)	26.64	−(0.00)	4.87	2.21	5.77	Consensus
*m*/*z* 267	+(1.00)	n/a	n/a	2.47	5.02	6.12	Nearest neighbor
*m*/*z* 273	+(1.00)	87.84	−(0.00)	5.02	2.36	5.66	Nearest neighbor
*m*/*z* 303	+(1.00)	n/a	n/a	4.21	2.10	7.01	Nearest neighbor
*m*/*z* 301	+(1.00)	n/a	n/a	5.02	2.36	7.18	Nearest neighbor
*m*/*z* 271	+(1.00)	n/a	−(0.00)	5.02	2.47	5.84	Nearest neighbor

n/a—not applicable.

**Table 4:** The efficiency comparison of DIA removal.

Removal Process	Media/Conditions	Removal	Reference
Photolysis	Milli-Q Water humic acids	103 ± 13.20 h 28 ± 12.20 h	[7]
Advanced membrane technology	Wastewater; GAC effluent UF-SW effluent	93.7% 90.4%	[44]
UV/chlorine simulated sunlight/chlorine	pH 7.0 phosphate buffer solutions	90.1% 72.4%	[66]
Photo-Fenton	Distilled water Fe(NO_3_)_3_	*t*_1/2_ 11.55 min	[67]
Photolysis	Wastewater effluent–solar irradiation River water–xenon lamp radiation	*t*_1/2_ 102.1 day 540.2 min	[62]
Photocatalysis	TiO_2_ MilliQ Pilot scale	*t*_1/2_ 2.56 h 1.01 h	This work

## Data Availability

Data will be made available upon request.

## Supplementary Materials

The following supporting information can be downloaded at: https://www.mdpi.com/article/10.3390/nano15110827/s1, Figure S1: Mass spectrum of diazepam; Figure S2: Mass spectrum of DP-1 with *m*/*z* = 273; Figure S3: Mass spectrum of DP-2 with *m*/*z* = 301; Figure S4: Mass spectrum of DP-3 *m*/*z* = 303; Figure S5: Mass spectrum of DP-4 with *m*/*z* = 271; Figure S6: Mass spectrum of DP-5 with *m*/*z* = 267.
