# Supplementary material for: Diazepam Photocatalytic Degradation in Laboratory- vs. Pilot-Scale Systems: Differences in Degradation Products and Reaction Kinetics
Source: Nanomaterials (Basel). 2025 May 29;15(11):827. doi: 10.3390/nano15110827 (PMC12158053; doi:10.3390/nano15110827)
Supplement: Supplementary file 1 [file nanomaterials-15-00827-s001.zip › nanomaterials-3636974-supplementary.pdf]

Supplementary Material Information

Diazepam Photocatalytic Degradation in Laboratory- vs. Pilot-Scale Systems: Differences in Degradation Products and Reaction Kinetics

Kristina Tolić Čop [1,*], Mia Gotovuša [1], Dragana Mutavdžić Pavlović [1,*], Dario Dabić [2] and Ivana Grčić [3]

[1] Faculty of Chemical Engineering and Technology, University of Zagreb, Trg Marka Marulića 19, 10000 Zagreb, Croatia; mgotovusa@fkit.unizg.hr

[2] Croatian Meteorological and Hydrological Service, Ravnice 48, 10000 Zagreb, Croatia; ddabic@dhz.hr

[3] Faculty of Geotechnical Engineering, University of Zagreb, Hallerova aleja 7, 42000 Varaždin, Croatia; igrcic@gfv.hr

* Correspondence: ktolic@fkit.unizg.hr (K.T.Č.); dmutavdz@fkit.unizg.hr (D.M.P.)

List of figures:

Figure S1. Mass spectrum of diazepam.

Figure S2. Mass spectrum of DP-1 with *m*/*z* = 273.

Figure S3. Mass spectrum of DP-2 with *m*/*z* = 301.

Figure S4. Mass spectrum of DP-3 with *m*/*z* = 303.

Figure S5. Mass spectrum of DP-4 with *m*/*z* = 271.

Figure S6. Mass spectrum of DP-5 with *m*/*z* = 267.

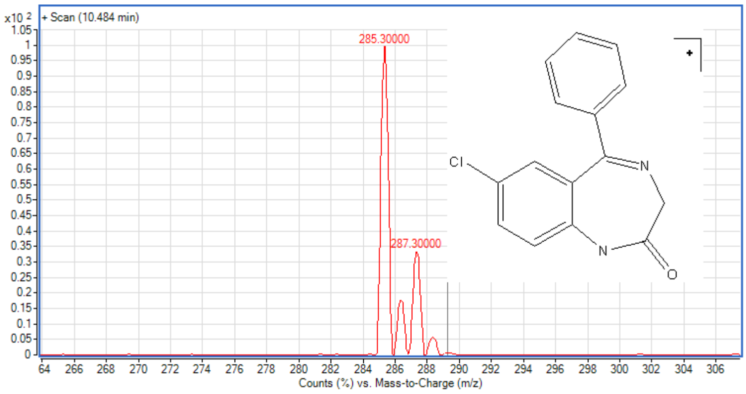

Figure S1. Mass spectrum of diazepam.

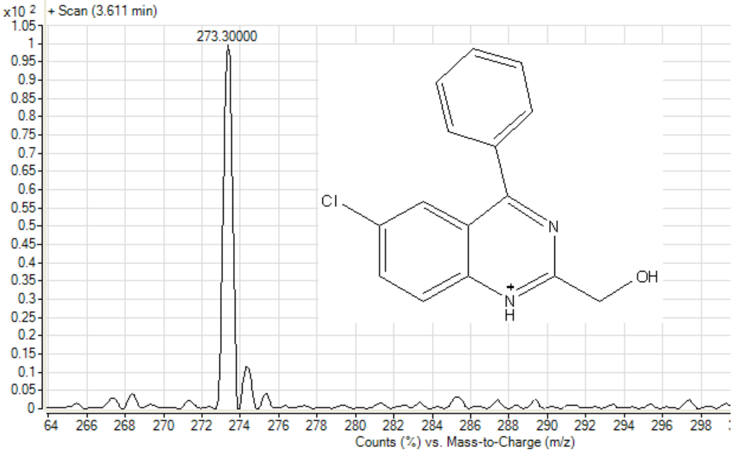

Figure S2. Mass spectrum of DP-1 with m/z = 273.

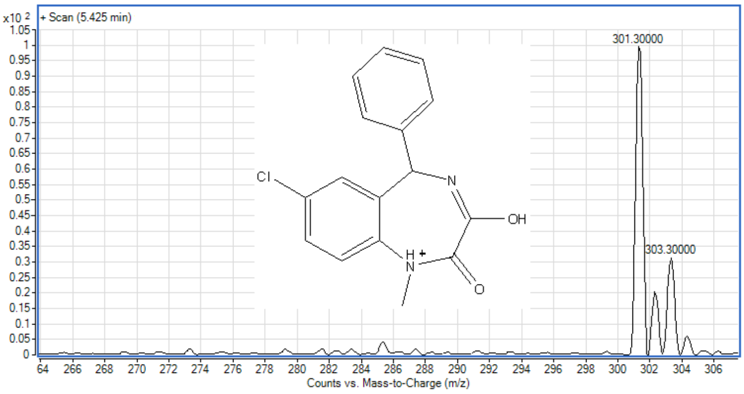

Figure S3. Mass spectrum of DP-2 with *m*/*z* = 301.

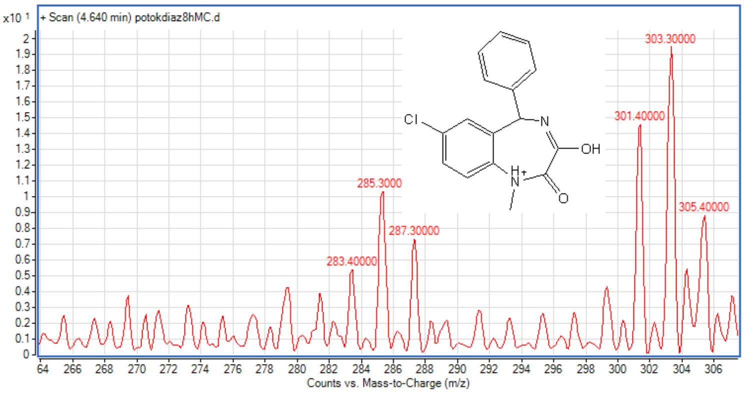

Figure S4. Mass spectrum of DP-3 with *m*/*z* = 303.

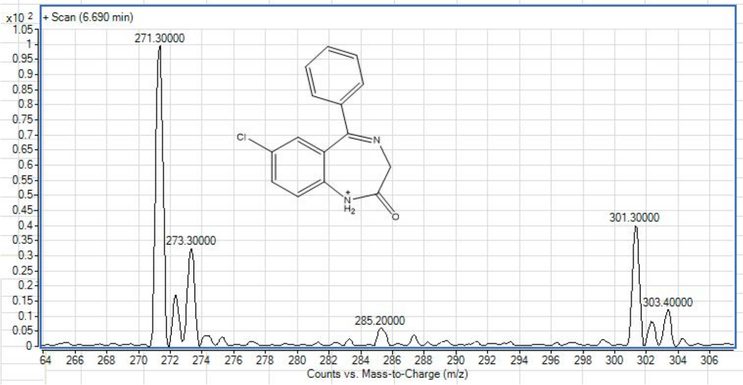

Figure S5. Mass spectrum of DP-4 with *m*/*z* = 271.

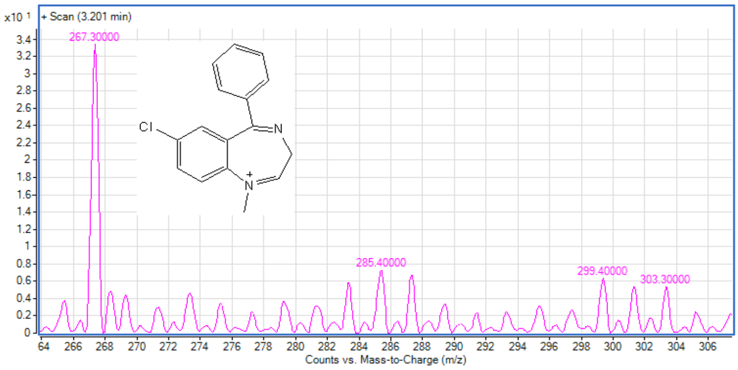

Figure S6. Mass spectrum of DP-5 with m/z = 267.
